# M1 But Not M0 Extracellular Vesicles Induce Polarization of RAW264.7 Macrophages *Via* the TLR4-NFκB Pathway *In Vitro*

**DOI:** 10.1007/s10753-020-01236-7

**Published:** 2020-04-22

**Authors:** Yulong Shi, Peng Luo, Weikang Wang, Klemens Horst, Felix Bläsius, Borna Relja, Ding Xu, Frank Hildebrand, Johannes Greven

**Affiliations:** 1 Department of Trauma and Reconstructive Surgery, RWTH Aachen University Hospital, Pauwelsstraße 30, 52074 Aachen, Germany; 2 Department of Radiology and Nuclear Medicine, Head of Experimental Radiology, University Hospital Magdeburg, Leipziger Str. 44, 39120 Magdeburg, Germany

**Keywords:** M1 macrophages, RAW264.7 macrophages, extracellular vesicles, polarization, NF-κB pathway

## Abstract

In response to different stimuli (*e.g.*, infections), naive macrophages polarize into M1 macrophages, which have the potential to secrete numerous pro-inflammatory cytokines and extracellular vesicles (EVs). EVs are important mediators of intercellular communication. *Via* horizontal transfer, EVs transport various molecules (*e.g.*, proteins, DNA, and RNA) to target cells. This *in vitro* study elucidated that M1-EVs from macrophages induced by interferon-γ (IFN-γ) and lipopolysaccharide (LPS) 24 h (M1), but not M0-EVs from untreated macrophages (M0), shifted M0 into M1 phenotype *via* activating the nuclear factor-κB pathway. The characteristics of these EVs were assessed by transmission electron microscopy (TEM), nanoparticle tracking analysis (NTA), and a western blot assay. RAW 264.7 cells were incubated with M1-EVs (experimental group) or PBS (sham group) or M0-EVs (control group) for 24 h. The viability, change of shape, and phenotype differentiation of the macrophages were identified by a 3-(4,5-dimethylthiazol-2-yl)-2,5-diphenyltetrazolium bromide (MTT) assay, flow cytometry, and immunofluorescence staining. The TLR4-NFκB pathway of RAW264.7 macrophages was assessed by a western blot assay. M1-EVs but not M0-EVs were incorporated by the RAW264.7 cells and directly induced polarization of RAW264.7 macrophages to M1 macrophages. This polarization was demonstrated by significant upregulation of the M1 macrophage marker CD86 in the experimental group (49.93 ± 5.0%) as compared with that in the control and sham groups (1.22% and 1.46%, respectively) and significant upregulation of iNOS in the experimental group (75 ± 5.0%) as compared with that in the control and sham groups (0%). Furthermore, cell viability was higher (1.3 times) in the experimental group as compared with that in both the sham and control groups. The regulatory mechanism of M1-EVs on RAW 264.7 macrophages polarization and activation was triggered by the activation of the TLR4-NFκB signaling pathway. Based on our observations, we conclude that M1-EVs play an important role in the M1 macrophage auto-polarizing loop. These data clearly demonstrate an important role for macrophage-derived EVs in cellular differentiation. Further studies are needed to elucidate the potential of these EVs in the modulation of inflammatory stimuli.

## INTRODUCTION

Macrophages represent an essential component of innate and acquired immune systems [1, 2]. Macrophage polarization and function are largely driven by tissue-derived and pathogenic stimuli, which help macrophages to adapt to changing conditions and impose a suitable reaction [3]. Stimulation with lipopolysaccharide (LPS), interferon-γ (IFN-γ), nitric oxide, or pro-inflammatory cytokines (*e.g.*, interleukin-1, interleukin-6, and TNF-α) causes polarization of classically activated macrophages (M1 macrophages) to a pro-inflammatory phenotype [4, 5]. The role of NFκB as an essential transcription factor in macrophage activation and polarization to a pro-inflammatory phenotype is well known [6, 7]. Activated M1 macrophages have the potential to induce high levels of pro-inflammatory cytokines, which can affect other tissues/cells and, in turn, activate and polarize additional macrophages, thereby amplifying the immune response [5]. Excessive magnification of pro-inflammatory processes can result in an unbalanced immune reaction and remote organ damage and dysfunction [4].

EVs are shed by various cell types in response to diverse signals and function as essential mediators of intercellular communication [8, 9].EVs originate from the multivesicular endosomal cell compartment or are released directly *via* outward budding from the cell plasma membrane. Subsequently they are released into the pericellular environment, circulating blood and other body fluids. *Via* horizontal transfer, EVs transport various molecules (*e.g.*, proteins, DNA, and RNA) to recipient cells [8, 9]. In addition to their role in signal communication, EVs have a variety of other functions, such as mediating growth and phenotype differentiation of their target cells [10–12]. The content (*e.g.*, membrane surface receptors, cytokines, DNA, and RNA) of EVs depends on the origin of the donor cell, microenvironment, and triggers for their release [11, 12].

Macrophage-derived EVs have procoagulant and apoptotic effects [13] and have been implicated in the development and progression of various diseases. EVs secreted from LPS-stimulated monocytes induced increased expression of adhesion molecules and cytokine production of endothelial cells that was closely associated with NFκB activation [14]. Previous studies demonstrated that macrophage-derived EVs exerted effects on target cells *via* a paracrine mechanism, such as facilitating pulmonary smooth muscle proliferation in patients with HIV infection [15], accelerating neutrophil necroptosis following hemorrhagic shock [16], or mediating acute lung injury [17]. Macrophage-derived EVs induced monocytes to develop into naive macrophages [18] and exerted both pro-inflammatory and anti-inflammatory effects [19].

The effects of M1-EVs on RAW264.7 cells’ activation and polarization so far remain unclear. The below described first experiments dealing with transpolarization of RAW264.7 cells by M1-EVs lead to human cell experiments in the future. Obtained information on these effects could potentially aid early treatment interventions. The goal of the present study was to characterize the function of M1-EVs and their ability to mediate the differentiation of RAW264.7 cells into the inflammatory-related M1 macrophage type for the first time.

## MATERIALS AND METHODS

### RAW 264.7 Cell Culture and Stimulation for M1 Differentiation

RAW 264.7 (ATCC) cells commonly used to mirror naive macrophage cells with a well-known and stable background were grown in high glucose Dulbecco’s modified Eagle’s medium (4.5 g/L) containing sodium pyruvate and L-glutamine (Gibco), supplemented with 10% heat-inactivated FBS, 100 U/ml of penicillin, and 100 μg/ml of streptomycin at 37 °C under 5% CO_2_ and 90% humidity.

For M1 differentiation, the RAW264.7 cells were washed and then treated for 24 h with 100 ng/ml of LPS (Sigma-Aldrich, St. Louis, MO) and 20 ng/ml of IFN-γ (eBioscience, San Diego, CA, USA) in EVs-free culture medium (EVs contaminants in DMEM depleted by centrifugation at 20,000×*g* for 90 min). After incubation for 24 h, the medium was harvested. LPS in combination with IFN-γ was chosen regarding the publication of Murray et al. which gave the best results concerning M1 characteristics of RAW264.7-derived M1 macrophages [20].

### EVs Isolation

Briefly, the harvested medium was collected and subjected to ultracentrifugation [17, 21]. Subsequently, the cells, cell debris, and apoptotic bodies were removed by centrifugation at 300×*g* for 10 min, 2000×*g* for 15 min, and 5000×*g* for 15 min, sequentially. The EVs were then pelleted by further centrifugation at 20,000×*g* (Beckman Coulter Avanti J-26XP, High-Speed Centrifuge and JA-25.50 Fixed-Angle Aluminum Rotor) for 90 min at 4 °C. The resulting precipitant was collected, suspended in 1 ml of PBS, and then centrifuged at 20,000×*g* for 90 min. The EVs were collected and stored at − 80 °C until further use. The storage period did not exceed 2 weeks.

### Group Distribution

RAW 264.7 cells assumed as naive macrophages (M0 macrophages) were treated with M1-EVs (experimental group), PBS (sham group), or M0-EVs (control group).

### EVs Visualization and Identification

TEM and NTA were used to characterize the EVs population and define its size and morphology. Briefly, EVs were fixed with 1% glutaraldehyde in PBS, and a 20-μl drop of each sample was placed on a carbon-containing grid and incubated for 1 min at room temperature (20 °C) for electron microscopy. Then, 2% phosphotungstic acid was used to stain each sample for 2 min, followed by observation under a JEM-1200 EX electron microscope (JEOL, Japan).

To confirm EVs isolation, the particles were visualized by NTA (NanoSight 300), which uses light scattering and Brownian motion to measure the particle size and concentration. The protein biomarkers of discrimination of EVs origins (TSG 101, CD 63, histone H3, and GAPDH) were identified by a western blot.

### EVs Uptake Assay

To determine whether M1-EVs or M0-EVs could be taken up by RAW 264.7 macrophages, the M1-EVs or M0-EVs were labeled with wheat germ agglutinin (Alexa Fluor™ 594 conjugate), a red fluorescent dye that binds to lipid regions of EV membranes, and incubated for 30 min at 37 °C. The labeled EVs were washed three times with PBS and centrifuged at 20,000×*g* for 120 min. After incubation with the macrophages for 24 h, the uptake of the EVs was stopped by washing and fixation in 4% paraformaldehyde and mounted with DAPI (Thermo Fisher Scientific, Waltham, MA, USA). Images were obtained using a microscope (FSX-100; Olympus, Tokyo, Japan).

### Immunofluorescence Staining of iNOS for M1 Determination

Immunofluorescence staining of iNOS was performed to determine the differentiation of RAW264.7 macrophages into M1 macrophages. After the treatment of M1-EVs or M0-EVs with RAW264.7, the cells were fixed with 4% paraformaldehyde or ice-cold methanol for 10 min and washed with PBS three times. After permeabilization by 0.3% Triton X-100 for 20 min and blocking with 5% BSA for 1 h, the cells were incubated with 5 μg/ml of FITC Mouse Anti-iNOS (BD Biosciences) for 20 min at room temperature. After washing with PBS, the slides were mounted with medium containing DAPI (Invitrogen, USA) and observed under fluorescence microscopy.

### MTT Assay for Determination of Macrophage Viability

Cell viability was assessed using an MTT assay. Briefly, 0.1 volume of MTT (1 mg/ml) was added to the cell culture medium and incubated for 4 h. The medium was removed, and 100 μl of DMSO were added into each well, and the plate was gently rotated to completely dissolve the precipitation. The absorbance of MTT was measured at 450/620 nm using a BioTek Synergy (BioTek, Winooski, VT, USA).

### Flow Cytometry for Macrophage Subtype Analysis

M1 macrophages and RAW 264.7 cells were detected by immunophenotyping using monoclonal antibodies specific for F4/80-APC and CD86-FITC (BD Biosciences, San Jose, CA, USA). For immunophenotypic analysis, the macrophages were gently detached by a cell scraper, pipetted up and down into single cell solutions, and suspended at 2 × 10^6^/ml. The cell suspensions were incubated for 15 min with 10% goat serum, followed by incubation with antibody mixtures for 30 min on ice. The cells were then washed twice with PBS containing 2% FBS. Data were immediately acquired using the BD LSR II system (BD Biosciences, San Jose, CA) and FlowJo software (Tree Star, San Carlos, CA, USA).

### Western Blotting for M1-EVs Characterization and Analysis of the M1 Differentiation-Related Pathway

Equal amounts of total proteins from the EVs samples or cell lysates were separated on 10% SDS-PAGE gels and then transferred onto PVDF membranes (PerkinElmer, USA). After blocking for 2 h at room temperature with 5% skimmed milk in trimethyl benzene sulfonyl tetrazole buffer, the membranes were incubated overnight at 4 °C with gentle shaking with antibodies against CD 63 (1:2000, Thermo Fisher Scientific), GAPDH (1:2000, rabbit, Thermo Fisher Scientific), TSG101 (1:500, rabbit, Thermo Fisher Scientific), histone H3 (1:1000, rabbit, Abcam), TLR4 (2 μg/ml, rabbit, Thermo Fisher Scientific), and p-NFκB-p65 (1:1000, rabbit, Thermo Fisher Scientific). Then the membranes were incubated with secondary antibodies for 30 min at room temperature. Finally, the immunoreactive protein bands were visualized using an enhanced chemiluminescence reagent, followed by imaging on an electrophoresis gel imaging analysis system (DNR Bio-Imaging Systems, Israel).

### Statistical Analysis

All experiments were performed in triplicates with three different RAW 264.7 stocks. Data were expressed as the average ± SD. Statistically significant differences (*P* < 0.05) between two groups and among more than two groups were evaluated by Student’s *t* test and one-way ANOVA with Tukey’s *post hoc* test, respectively.

## RESULTS

### Confirmation and Characterization of M-EVs

**Fig. 1:**
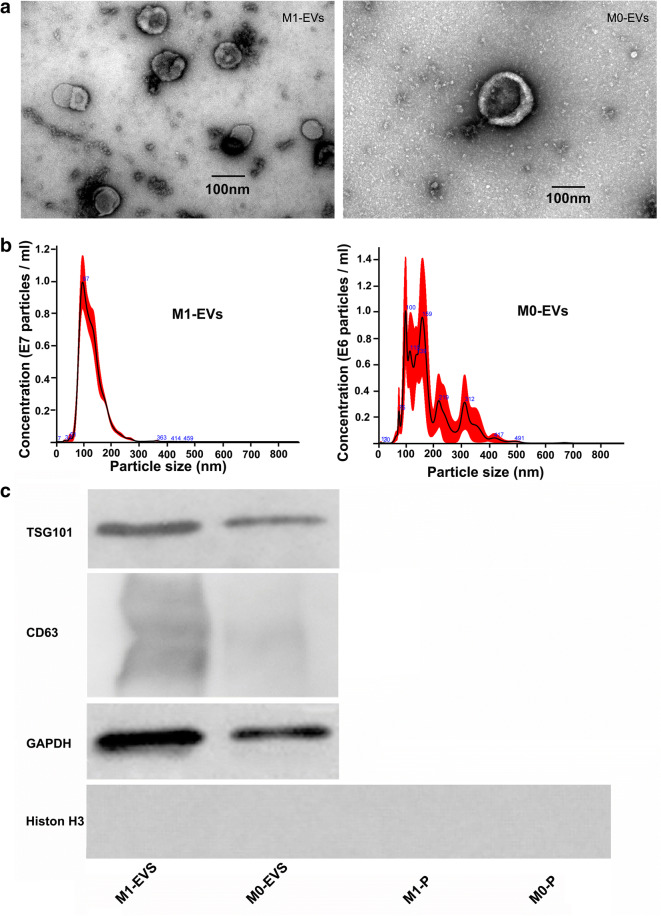
Characterization of M-EVs. **a** Electron micrograph of M1-EVs and M0-EVs shows a circular and double-membrane structure. **b** The size distribution and total number of EVs from M1 and M0 were measured by nanoparticle tracking analysis. **c** The presence of the EVs protein markers Tsg101, CD63, histone H3, and GAPDH; M1-P and M0-P stand for pellet from the 5000×*g* spin of M1-EVs and M0-EVs group, respectively.

M1 macrophages were successfully cultivated by polarization from RAW264.7 cells following stimulation with LPS and IFN-γ. Transmission electron microscopy analysis of the isolated revealed that both M1-EVs and M0-EVs showed similar typical double-membrane structures and a spheroid shape (Fig. 1a). The NTA assay showed that the purified M1-EVs samples ranging from 70 up to 400 nm in diameter had a mean size of around 126.2 ± 2.4 nm (SD, 41.9 ± 1.1 nm) and a concentration of 7.50 × 10^8^ ± 8.74 × 10^7^ particles/ml, with M0-EVs samples ranging from 70 up to 600 nm in diameter had a mean size of 150.6 ± 24.1 nm (SD, 49.9 ± 17 nm) and a concentration of 1.09 × 10^8^ ± 4.23 × 10^7^ particles/ml. The diameter of M1-EVs was more uniform (single diameter concentration peak at around 97 nm) and smaller than M0-EVs with several diameter concentration peaks. The concentration of M1-EVs was almost sevenfold compared with the M0-EVs (Fig. 1b). Concentrations used in the experimental settings were used accordingly. Figure 1 c shows enrichment of EVs containing the vesicle marker TSG 101, CD 63, and GAPDH (Fig. 1c). The apoptotic marker (histone H3) included in the western blot showed a negative result in the M1/M0-EVs and the pellet from the 5000×*g* spin of M1/M0-EVs groups.

### Incorporation of M1-EVs into RAW 264.7 Cells Induced Amoeboid Morphology

**Fig. 2:**
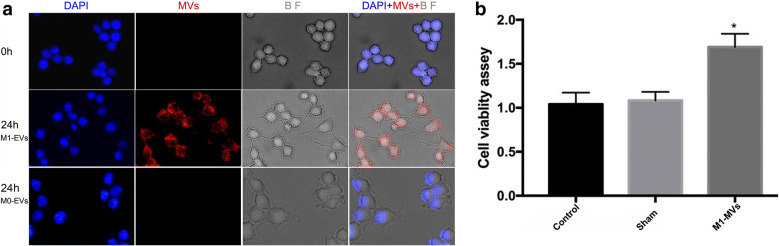
M1-EVs uptake by naive macrophages and upregulate macrophages viability. **a** showed that confocal microscopy images reveal the internalization of fluorescently labeled EVs into naive macrophages. Naive macrophages were incubated with red-labeled M1-EVs at 37 °C for 24 h and viewed by confocal microscopy. Cellular morphology (BF, gray) was visualized without fluorescence using differential interference contrast, blue DAPI indicated for macrophage nuclei, and red EVs. The shape of polarized macrophages changed over the time course of M1-EVs incorporated into the naive macrophages (BF, gray). Magnification, × 400. **b** revealed the results of cell viability assay on naive macrophages incubated with M1-EVs for 48 h analyzed by MTT. (*n* = 5, M1-EVs group *vs.* control group, *P < 0.001, compared with the sham and control group).

As shown by fluorescence microscopy, red wheat germ agglutinin signals were observed in the macrophages treated with wheat germ agglutinin-labeled M1-EVs (Fig. 2a). This finding indicated that M1-EVs were incorporated into RAW264.7 macrophages after 24 h of incubation. In a bright field scan, naive macrophages incubated with M1-EVs changed from an oval or ramified morphology to an amoeboid shape, with a larger cell area and longer cell diameter. The red immunofluorescence signal was not observed in macrophages co-cultured with M0-EVs, and the morphology of macrophages remained still, which elucidated that M0-EVs could not be transported into naive macrophages.

### RAW 264.7 Macrophage Viability After EVs Incubation

As compared with that of the RAW 264.7 cells in the control and sham groups, macrophage viability activity significantly increased in the experimental group (1.3 times higher) (*p* < 0.0001, *n* = 5, Fig. 2b), which was consistent with the observed changes in macrophage shape in the EVs uptake assay.

### Modulation of RAW 264.7 Macrophage Polarization by M1-EVs

**Fig. 3:**
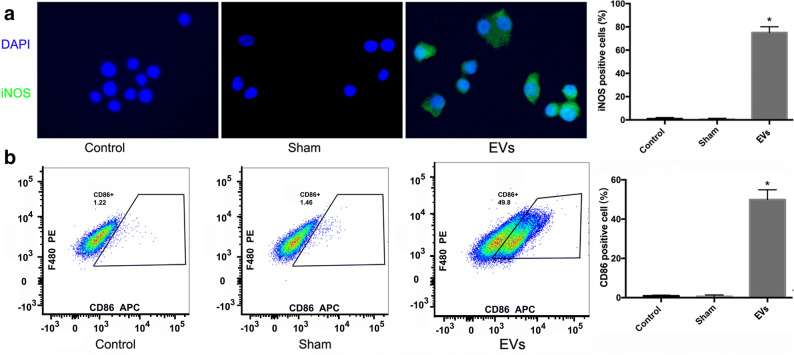
M1-EVs treatment for 24 h induced expression of iNOS and CD86 in RAW 264.7 macrophages. **a** showed that M1-EVs upregulated iNOS in RAW 264.7 macrophages; naive macrophages were treated with PBS (sham group), M1-EVs (EVs group), or M0-EVs treatment (control group), respectively. Magnification, × 400. (*n* = 3) *P < 0.0001, compared with the sham and control group. **b** showed flow cytometry plots of macrophage marker CD86; naive macrophages were treated with PBS (sham group), M1-EVs (EVs group), or M0-EVs treatment (control group), respectively. After 24 h, the presence of macrophage expressing CD86 in EVs group increased sharply, and the percent of surface marker CD86 expression level in the control and sham groups almost remained unchanged. Quantification of surface marker expression was presented as mean + SEM (*n* = 3). *P < 0.0001, compared with the sham and control group.

The expression of iNOS and CD 86 as detected by immunofluorescence staining (Fig. 3a) and quantified by FACS (Fig. 3b) was significantly increased in the experimental group as compared with that in the control and sham groups. Incubation with M1-EVs resulted in increases of 75 ± 5.0% in iNOS and 49.93 ± 5.0% in CD 86-positive stained RAW264.7 cells, as shown by histology and flow cytometry. These findings indicated that the macrophages in the experimental group had differentiated into M1 macrophages, whereas those in the control and sham group had not (*i.e.*, they were naive macrophages).

### M1-EVs Activated the TLR4-NFκB Signal Pathway of RAW 264.7 Cells

**Fig. 4:**
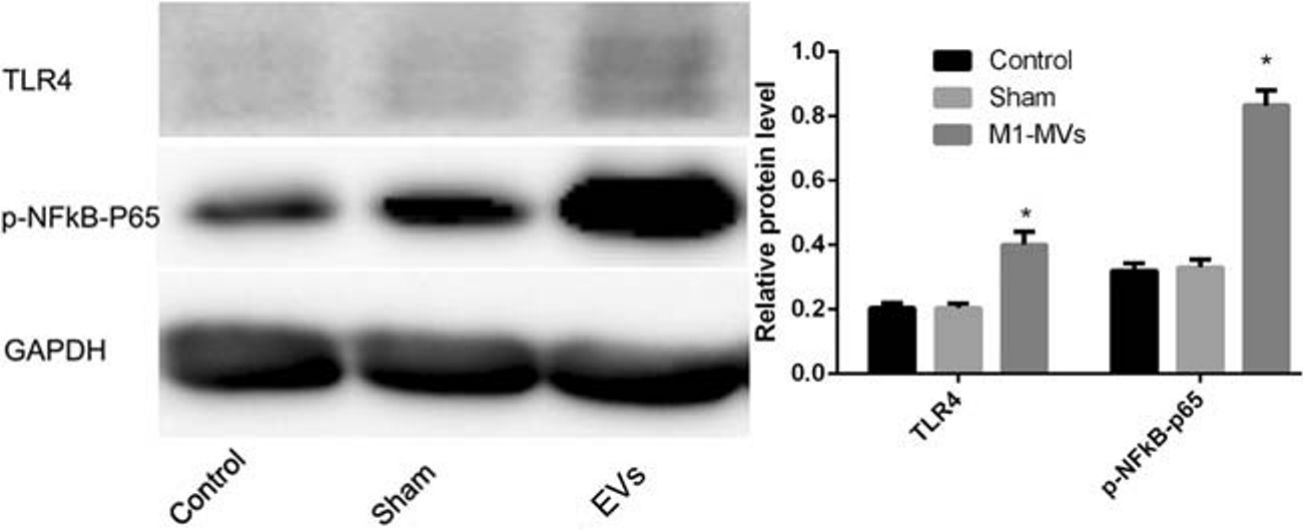
M1-EVs increase TLR4-NFκBp65 in naive macrophages, TLR4-NFκBp65 expression assessment in naive macrophages treated with PBS (sham group), M1-EVs (experiment group), or M0-EVs treatment (control group) for 24 h by western blot, respectively. The amount of TLR4 and p-NFκBp65 protein levels were normalized by GAPDH (*n* = 3). *P < 0.01, compared with the sham and control group.

As compared with the macrophage protein levels of TLR4-NFκB-p65 in the control and sham groups, those in the experimental group increased significantly (Fig. 4). This finding suggested that the effects of M1-EVs on viability and phenotypic differentiation of RAW264.7 macrophages seemed at least partly mediated by activation of the TLR4-NFκB-p65 signaling pathways.

## DISCUSSION

Macrophages play a critical role in the development, progression, and resolution of inflammation [1, 2]. M1 phenotype (pro-inflammatory) macrophages arise as a result of classical activation *via* stimulation of TLR receptors through appropriate ligands, such as microbial stimuli (*e.g.*, LPS) alone or in conjunction with cytokines (*e.g.*, IFN-γ and TNF-α) [4, 22]. M1 macrophages produce inflammatory mediators, such as IL-6, nitric oxide, and TNF-α, thereby significantly contributing to the immune response [22]. These mediators can be transferred and encoded into EVs, which, in turn, can be incorporated into recipient cells and directly stimulate these cells. Several studies have demonstrated that macrophage-derived EVs can exert pro-inflammatory effects on various cell types such as naive monocytes/macrophages [13–19].

Our study demonstrated for the first time that EVs from polarized M1 macrophages but not EVs from quiescent macrophages significantly promoted the viability of RAW264.7 cells and stimulated these to polarize into M1 macrophages like cells. M1-EV-mediated TLR4-NFκB signaling played a vital role in this polarizing process. Applying sequential centrifugation, the EVs populations less than 1 μm in size were successfully isolated which were free of detached cells, apoptotic bodies, or platelets. Detection of the markers TSG101, CD63, histone H3, and GAPDH and the distinct peaks observed in the NTA underline that the obtained results were dependent on EVs uptake only and not related to debris contamination. These results indicated that EVs may not only mediate cell-to-cell communication but also represent a key source for paracrine stimulation.

As shown in previous research, an RAW264.7 murine macrophage cell line released EVs following stimulation with LPS or polyinosinic-polycytidylic acid or stimulation with ligands of TLR4 and TLR3 [23]. We also demonstrated that EVs released at very a low level under physiological conditions (naive macrophages shedding) and markedly enhanced in pathologic status (M1 macrophages secreting). The varied and larger M0-EVs with several diameter concentration peaks differentiated into steadier M1-EVs, which may contribute to transportation under pathologic circumstance.

In accordance with our results, previous studies provided evidence that macrophages and monocytes have the potential to produce EVs for intercellular communication to maintain homeostasis and immune cell production, as well as to induce genetic and phenotypic changes of target cells [13, 15, 16]. According to one study, EVs derived from macrophages acquired from the systemic circulation polarized naive monocytes into naive macrophages [18]. Furthermore, EVs derived from systemically acquired monocytes activated both human monocytes and monocyte-derived macrophages [19]. However, in these studies [18, 19], the EVs were derived from circulating monocytes/macrophages originating from peripheral human blood. Nevertheless, they incubate the isolated cells with EVs of their own as an autocrine effect. In contrast to the previous studies and to avoid co-culturing with EVs originated from systemically circulated cells, we co-cultured RAW264.7 macrophages with M1-EVs that were taken from previously *in vitro* polarized M1 macrophages differentiated by LPS and IFN-γ stimulation. This setup mimics the effect of paracrine activation and naive macrophage polarization by tissue-resident M1 macrophages in the early pro-inflammatory phase.

For the first time, we showed that polarization of naive macrophages was significantly activated by M1-EVs. Such activation can be inferred based on “stretching” of the arms (pseudopodia) of the originally round-shaped naive macrophages. These arms are thought to be induced to engulf antigens and enable the secretion of increased amounts of cytokines due to a larger cell surface [24]. Although these morphological changes might not be sufficient to infer macrophage functional behavior, their role in macrophage activation and function is accepted [24, 25].

In addition to the morphological changes observed herein, polarization of naive macrophages by M1-EVs into M1 macrophages in our study was also indicated by activation of the TLR4-NFκB signaling pathway and an associated increase in iNOS and CD86 expression. Previous research reported that alveolar macrophages were activated (*e.g.*, release of pro-inflammatory cytokines) *via* the TLR4-NFκB signal pathway when subjected to mesenteric lymph exosomes from hemorrhagic shock rats [26]. In addition, the TLR4-NFκB pathway of human monocytes and monocyte-derived macrophages was induced when stimulated by EVs derived from calcium ionophore A23187-stimulated monocytes [19]. Various studies have also demonstrated that activation of the transcription factor TLR4-NFκB was associated with enhanced expression of iNOS and CD86, typical markers of M1 macrophages [7, 27, 28]. These results are in line with those of our study. However, these significant effects of M1-EVs on quiescent macrophages were not discovered in the static macrophages treated with M0-EVs. The possible reasons may be (1) the concentration of EVs released by untreated macrophages was very low which determines the amount applied in stimulating macrophages, (2) the trigger of M0-EVs exerting on static macrophages is the condition they are under, and (3) the heterogeneity between the M1-EVs and M0-EVs, which fails M0-EVs effectively shifting naive macrophages into M1 phenotype. A prior study has demonstrated that M0-EVs could not activate endothelial cells in M1-EVs-like fashion by triggering the NFκB pathway [29]. Further studies need to elucidate the underlying detail mechanisms of M1-EVs on macrophages phenotypic shifting, such as the specificity of macrophages binding and absorbing EVs.

## CONCLUSION

To our knowledge, this is the first study to uncover the function of pro-inflammatory macrophage-derived EVs in M0 macrophage differentiation. The identified feedback mechanism may be an innate response, which activates the local immune system. As an excessive inflammatory response may harm the host through maladaptive release of endogenously generated inflammatory compounds, analyzing the content of EVs may aid predictions of the severity of inflammation and modulation of systemic inflammation. Although limited to an *in vitro* cell model, these data expand our knowledge of the important role of macrophage-derived EVs in cellular homeostasis.
